# Long-Term Safety and Efficacy of Percutaneous Left Atrial Appendage Closure with the LAmbre Device

**DOI:** 10.1155/2020/6613683

**Published:** 2020-12-21

**Authors:** Guangji Wang, Bin Kong, Yu Liu, He Huang

**Affiliations:** ^1^Department of Cardiology, Renmin Hospital of Wuhan University, Wuhan, Hubei, China; ^2^Cardiovascular Research Institute of Wuhan University, Wuhan, Hubei, China; ^3^Hubei Key Laboratory of Cardiology, Wuhan, Hubei, China

## Abstract

**Background:**

Left atrial appendage closure (LAAC) using the LAmbre device has been associated with prevention of stroke in patients with nonvalvular atrial fibrillation (AF). Here, we interrogated the long-term safety and efficacy of using the LAmbre device in percutaneous LAAC.

**Methods:**

We analyzed 56 records of patients with nonvalvular AF undergoing LAAC procedures with the LAmbre device. We collected and analyzed the data to define the safety and efficacy of the LAmbre device implantation.

**Result:**

The LAAC was successfully occluded in the 56 patients. Our data showed no serious residual leak or pericardial effusion occurred during the perioperative period. At a mean follow-up of 37.8 ± 23.5 months, there were 7.1%, 3.6%, and 3.6% rates of death, stroke, and device-related thrombus, respectively. There were no cases of severe residual leak or systemic embolism.

**Conclusion:**

Taken together, we demonstrate that execution of LAAC with the LAmbre device has high procedural success and prevents AF-related stroke. However, further large-scale trials might be required to confirm our findings.

## 1. Introduction

Atrial fibrillation (AF) is the most common sustained arrhythmia, which is associated with an increased risk of stroke [1]. AF-induced stroke events are more severe than non-AF stroke events and are associated with a higher risk of morbidity and mortality [2]. Currently, oral anticoagulation (OAC) remains the most effective way to prevent stroke in patients with AF. However, for patients contraindicated with OAC or those who refuse OAC therapy, left atrial appendage closure (LAAC) with Watchman (Boston Scientific, Natick, Massachusetts) or Amplatzer Cardiac Plug (ACP; St. Jude Medical, Saint Paul, Minnesota) presents feasible alternatives [3–9].

The LAmbre (Lifetech Scientific, Shenzhen, China) left atrial appendage (LAA) occluder is a novel system, especially designed for LAA closure in cases that present morphological difficulties [10]. Previous studies have confirmed that LAAC with the LAmbre is safe and effective in the prevention of thromboembolic events [11–13], but the effectiveness and the safety were similar to Watchman and ACP [14]. However, most of the clinical studies on the LAmbre LAA occluder were followed up for 1 year. To date, there are no data on the long-term safety and efficacy of LAAC with the LAmbre occluder in patients with AF. Here, we aimed to evaluate the long-term effect of the LAmbre LAA occluder in patients with AF.

## 2. Method

### 2.1. Study Population

A retrospective, single-center study was performed in 56 patients with nonvalvular AF, who underwent LAAC with the LAmbre device between March 2014 and June 2020. We included patients with nonvalvular AF, aged 18 years and above, and a CHA_2_DS_2_-VASc Score ≥2. The patients were contraindicated to long-term oral anticoagulants (OACs) or refused OAC therapy. We excluded those with severe valvular disease or abnormal cardiac structure, left atrial or LAA thrombosis confirmed by transesophageal echocardiogram (TEE), pregnancy or breastfeeding, and left ventricular ejection fraction (LVEF) < 30%, as well as significant pericardial effusion. This study was approved by the Human Subject Ethics Committee of Renmin Hospital, Wuhan University. All patients gave signed informed consent.

### 2.2. LAmbre LAAC

**Figure 1:**
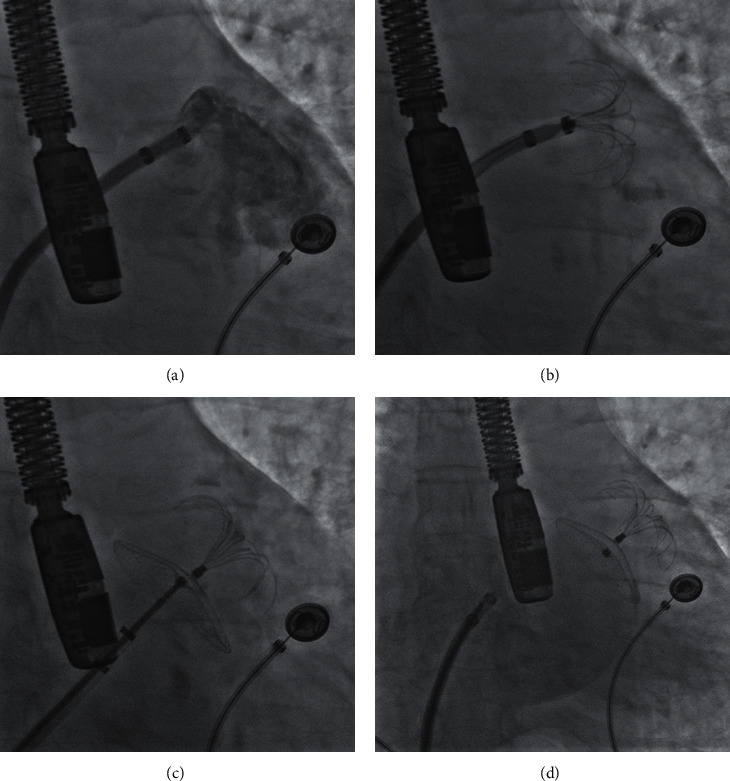
The procedure of LAA closure with the LAmbre device. (a) LAA angiogram assessment of the left auricle size. (b) The deployment of the umbrella. (c) The deployment of the cover. (d) Left atrial angiogram was performed after the release of the device to check for LAA sealing.

The LAmbre LAAC was performed either under local or general anesthesia, aided by the TEE and X-ray fluoroscope. Besides, transseptal puncture was performed under the guidance of the TEE. Selective LAA angiogram was performed to understand the shape, size, and adjacent relationships, and the TEE was used to measure the diameter and depth of the LAA. The size of the LAA occluder was chosen based on the data from the LAA angiogram and TEE measurements. LAA angiogram and TEE were also performed to determine the location and placement of the occluder. In addition, a gentle tug test was performed under fluoroscopy to ensure device stability. The occluder was completely retrieved if the position, sealing effect, and stability were not satisfying (Figure 1).

### 2.3. Postsurgical Anticoagulation

The double antiplatelet therapy (aspirin plus clopidogrel) was performed for 3 months after LAAC, followed by TEE examination 3 months later. In cases where there was no significant residual shunt, the double antiplatelet treatment was changed to either aspirin or clopidogrel for long-term treatment.

### 2.4. Follow-Up

In the first year, the patients were monitored at 1, 3, 6, and 12 months after LAAC. Long-term follow-up was performed using phone or mail survey assessment.

### 2.5. Endpoints

The study endpoints included severe perioperative complications and serious adverse events (SAE) during the follow-up. The severe perioperative complications and SAE were defined as death, stroke, cardiac effusion, major bleeding, severe vascular complications, thrombosis with device, systemic thromboembolism, and device dislocation.

### 2.6. Statistical Analysis

Categorical variables were expressed as frequencies and percentages. On the other hand, continuous variables were presented as mean ± SD.

## 3. Results

### 3.1. Base Characteristics

A total of 56 patients (66.6 ± 8.4 years; 23 females) were enrolled in this study (Table 1). The mean CHA_2_DS_2_-VASc Score was 3.7 ± 1.3, while the mean score for HAS-BLED was 2.3 ± 0.9. Our analysis revealed that 26 (46.4%) patients had a history of prior stroke/TIA, while 21 (37.5%) had a previous coronary artery disease. In addition, 46 (82%) patients had previous hypertension and 8 (14.3%) had a history of diabetes while 10 (17.9%) and 46 (82.1%) patients had a paroxysmal and a nonparoxysmal AF, respectively.

### 3.2. Procedural Characteristics

The success rate for the LAmbre device implantation was 100%. The mean LAA length was 29.2 ± 6.5 mm, LAA orifice diameter was 27.6 ± 5.2 mm, and the LAA landing zone diameter was 22.6 ± 4.4 mm. Our TEE analysis showed that there was 1 (1.8%) patient with <1 mm residual flow and there were 5 (8.9%) patients with 1–3 mm residual flow. There were 3 cases of periprocedural complications associated with slight pericardial effusion. There was however no cases of death, stroke, major bleeding, major vascular complication, thrombosis with device, and device dislocation (Table 2).

### 3.3. Patient Follow-Up Data

As shown in Table 3, the mean follow-up time was 37.8 ± 23.5 months. During the follow-up period, there were 4 (7.1%) deaths. 3 cases were noncardiac death at 3 months, 36 months, and 49 months after the procedure, while the other was cardiac arrest at 46 months after the procedure. There were 2 (3.6%) cases of ischemic stroke during the follow-up period. The TEE analysis showed that device thrombosis occurred in 2 (3.6%) patients. Besides, TEE reexamination revealed thrombotic dissolution after anticoagulant therapy. Two (3.6%) patients had <1 mm residual flow, while 1 (1.8%) had 1–3 mm residual flow.

## 4. Discussion

Here, we demonstrate that LAAC with the LAmbre device has a high success rate. In addition, long-term follow-up results robustly associate the LAmbre LAAC with good clinical outcomes in the prevention of stroke.

Many clinical studies have associated LAAC with reduction in stroke [3–13]. While the LAAC device therapy seems promising, the incidence of treatment-related complications remains undefined. For instance, the PROTECT-AF and CAP and PREVAIL studies showed that the success rate for the Watchman device implantation was 91.3% and 95%, respectively [3–5]. With the improvement in the implantation techniques, the Watchman device implantation success rate increased to 98.5% in the EWOLUTION study [7]. On the other hand, initial European and Asia-Pacific experience suggested that the ACP device implantation success rate was 96% (132/137) and 95% (19/20), respectively [8, 15]. A multicenter study associated the use of the ACP device with 97.3% success rate [9]. In this study, unlike the Watchman and ACP devices, we show 100% success with the use of the LAmbre device. Similarly, the initial European registration studies reported 100% success rate with the LAmbre device implantation [11]. In addition, a prospective, multicenter clinical study suggested that the LAmbre device implantation success rate was 99.4% [13]. The high implantation success rate demonstrated with the use of the LAmbre device may be due to its unique design. The LAmbre device is shorter in size and requires less depth for the LAA. In addition, there are diverse sizes in the LAmbre device, which makes it more suitable for the special shape of the LAA.

Pericardial effusion is the most common perioperative complication of LAAC with the Watchman device, whose severity harbors fatal risks [3]. In the PROTECT-AF study, the incidence of serious pericardial effusion was 4.8% (22/463), of which 15 were treated with pericardiocentesis, while the rest underwent surgical intervention [3]. On the other hand, in the CAP study, the incidence of serious pericardial effusion in patients with Watchman occluder implantation was only 2.2% (10/460) [4]. The EWOLUTION registry reported that the pericardial effusion only occurred in 5 patients (0.5%, 5/1004) [7]. Initial European experience reported 3.5% (5/143) cases of serious pericardial effusion after ACP implants [8]. However, the severe pericardial effusion with ACP occluder implantation was dependent on the operator's experience. In other ACP registries, no serious pericardial effusion was observed [8, 9, 15]. The occurrence of pericardial effusion was lower in LAmbre registries compared to either the ACP or Watchman registries. In the LAmbre series of 153 patients, serious pericardial effusion occurred only in 3 patients (2.0%) [13]. Perioperative pericardial incidences were neither reported in the LAmbre series of 60 patients nor in the series of 30 patients [11, 12]. However, 2 late pericardial effusions were reported in the series of 60 patients [12]. In our study, no serious pericardial effusion occurred.

Other typical LAAC perioperative complications, such as air embolization, cardiac perforation, major bleeding, device dislocation, or device embolization, were not observed. This might be attributed to the fact that the LAmbre device includes a specially designed hook umbrella with the ability to recapture, retrieve, redeploy, and remain stable during deployment. In addition, the transport sheath of the LAmbre occluder is smaller (8–10 French) than that of Watchman or ACP occluders (14 French).

Residual flow is a common complication in LAAC. The factors influencing the occurrence of residual flow following LAAC are still unclear. Some studies suggested that it may be related to the morphology and type of the LAA, the surrounding structure, and the compression ratio of the occluder [16–19]. EWOLUTION registry showed >5 mm residual flow in 7 patients (0.7%) and ≦5 mm residual flow in 78 patients (7.9%) following Watchman device implantation [7]. Urena et al. [20] studied 52 patients enrolled in 7 centers and found that the incidence of <3 mm residual flow immediately and at 6-month follow-up was 13.5% and 16.2%, respectively, following ACP device implantation. The rate of residual flow in the LAmbre device appears to be higher than the ones reported for the ACP and Watchman devices. For instance, Chen et al. [11] reported that there were 5 (20%) patients with a residual flow of 2 mm, 3 (12%) patients with a residual flow of 3 mm, and 1 (4%) patient with a residual flow of 4 mm, during the follow-up period. On the other hand, Park et al. [12] showed that the rate of <5 mm residual flow was 14/57 (24.6%), 11/54 (20.4%), or 15/36 (41.7%), at 1, 6, and 12 months of follow-up, respectively. Besides, a residual flow of ≧5 mm was observed in 3/60 (5%) patients at the first month of follow-up. In a multicenter study, the residual flow of <1 mm was 1.3% (2 cases), while the residual flow of 1–3 mm and >3 mm was 13.3% and 0.7%, respectively, immediately after the procedure [13]. The reason of high incidence of residual flow after LAAC with the LAmbre device may be related to the operator's operating experience. Compared with Watchman and ACP occluders, the LAmbre occluder is in less clinical use. The incidence of residual flow may decrease with the accumulation of operator experience. Compared to these studies, the incidence of residual flow in our study was relatively lower. In our study, a residual flow of <1 mm or 1–3 mm was observed in 1.8% or 8.9% patients, respectively, immediately after the procedure with the LAmbre device. During the follow-up, 2 (3.6%) patients had a <1 mm residual flow, while 1 (1.8%) patient had residual flow of 1–3 mm. However, to date, the relationship between residual flow and stroke remains controversial.

Device-related thrombus (DRT) is a common medium- and long-term complication after LAAC. A previous study demonstrated that the DRT  is associated with a higher rate of stroke and systemic embolism [21, 22]. A 1-year follow-up data of the EWOLUTION trial reported that the incidence of DRT  with the Watchman device was 3.7% [23]. In the ASAP study (ASA plavix feasibility study with Watchman left atrial appendage closure technology), there were 6 cases (4%) of DRT [24]. In addition, Saw et al. [25] reported the incidence of DRT in the ACP device trial to be 3.2%. The multicenter experience of LAAC with the ACP also reported a 4.4% (28/632 patients) DRT incidence [9]. However, the frequency of DRT after LAAC with LAmbre is marginal [8, 9]. A multicenter clinical study reported 2 patients (1.3%) with DRT following LAAC with LAmbre. Our present data showed 2 patients (3.6%) with DRT. However, the observed rate of ischemic stroke in our study was higher than those reported in other LAAC trials [9, 13, 23]. Lucas et al. observed a 1.1% rate of ischemic stroke at a 1-year follow-up in a Watchman device trial [23]. Similar data were obtained in the ACP device trial [9]. A multicenter clinical study with the LAmbre device showed 1.3% ischemic stroke cases [13]. Here, we reported 2 patients (3.6%) with ischemic stroke, 1 of which terminated the use of oral antiplatelet drugs one year after the procedure. Therefore, adequate antithrombotic therapy with aspirin and clopidogrel is important to prevent thrombus formation after LAAC.

The mortality rate after LAAC varies greatly in different studies. A 1-year follow-up outcome data in the EWOLUTION trial reported a 9.8% mortality rate after LAAC with the Watchman device [23]. A 5-year mortality rate was 3.6% in the PREVAIL and PROTECT-AF trials following LAAC with the Watchman device [26]. In addition, a one-year all-cause mortality from multicenter data with the ACP was 4.2% [9]. Besides, a global prospective observational study reported a 2.1% mortality rate after LAAC with the ACP device [27]. Marian et al. observed a 16.6% mortality rate in a LAmbre device trial. In a series of 153 patients, the mortality rate after LAAC with the ACP device was 0.7% [13]. Here, while the observed mortality rate was 7.1%, none of the deaths were related to the procedure. The diverse mortality rate outcomes could be associated with the different ages of the included patients, as well as different underlying diseases and follow-up time.

## 5. Limitation

This study was conducted retrospectively, was based on a single center, and has a small sample size. Besides, the follow-up assessments for embolic events were detected based on the description of patients; therefore, the occurrence rate might be underestimated. Prospective, multicenter, randomized, and controlled clinical trials are therefore needed to further confirm the efficacy and safety of the use of the LAmbre device.

## 6. Conclusion

Taken together, we deduce that LAAC with the LAmbre device is associated with a low rate of stroke and bleeding events. Multicenter, large-scale, randomized, and controlled studies are needed to further verify the long-term safety and efficacy of the LAmbre device.

## Figures and Tables

**Table 1:** Baseline characteristics.

	*n* = 56
Age (years)	66.6 ± 8.4
Female, *n* (%)	23 (41.1)
BMI (kg/m^2^)	25.1 ± 3.2
Hypertension	46 (82.0)
Diabetes	8 (14.3)
Previous stroke/TIA	26 (46.4)
Coronary artery disease	21 (37.5)
Paroxysmal AF	10 (17.9)
Nonparoxysmal AF	46 (82.1)
CHA_2_DS_2_-VASc Score	3.7 ± 1.3
HAS-BLED Score	2.3 ± 0.9

BMI = body mass index; AF = atrial fibrillation.

**Table 2:** Periprocedural data.

	*n* = 56
LAA diameters	
LAA length (mm)	29.2 ± 6.5
LAA orifice diameter (mm)	27.6 ± 5.2
LAA landing zone diameter (mm)	22.6 ± 4.4
Number of LAA lobes	
Single lobe	42 (75)
Two lobes	12 (21.4)
Multiple lobes	2 (3.6)
Successful implantation	56 (100)
LAA leak	
Residual flow <1 mm	1 (1.8)
Residual flow 1–3 mm	5 (8.9)
Residual flow >3 mm	0
Procedure time (min)	60.1 ± 13.0
Contrast media (ml)	44.5 ± 13.7
Complications	
Death	0
Stroke	0
Pericardial effusion	0
Major bleeding	0
Major vascular complication	0
Thrombosis with device	0
Device dislocation	0

LAA = left atrial appendage.

**Table 3:** Clinical outcomes during follow-up.

	*n* = 56
Follow-up time (months)	37.8 ± 23.5
Death	4 (7.1)
Noncardiac death	3 (5.4)
Cardiac death	1 (1.8)
Ischemic stroke	2 (3.6)
Hemorrhagic stroke	0
Device thrombosis	2 (3.6)
Systemic thromboembolism	0
LAA sealing by TEE examination	
Residual flow <1 mm	2 (3.6)
Residual flow 1–3 mm	1 (1.8)
Residual flow >3 mm	0

LAA = left atrial appendage; TEE = transesophageal ultrasound.

## Data Availability

The data used to support the findings of this study are available from the corresponding author upon request.
